# Impact of Working Environment on Job Satisfaction: Findings from a Survey of Japanese Dental Hygienists

**DOI:** 10.3390/ijerph18063200

**Published:** 2021-03-19

**Authors:** Ayako Okada, Yuki Ohara, Yuko Yamamoto, Yoshiaki Nomura, Noriyasu Hosoya, Nobuhiro Hanada, Noriko Takei

**Affiliations:** 1 Department of Operative Dentistry, Tsurumi University School of Dental Medicine, Yokohama 230-8501, Japan; okada-a@tsurumi-u.ac.jp; 2 Tokyo Metropolitan Institute of Gerontology, Tokyo 173-0015, Japan; yohara@tmig.or.jp; 3 Japanese Dental Hygienists’ Association, Tokyo 169-0071, Japan; nori@pm-ms.tepm.jp; 4 Department of Endodontology, Tsurumi University School of Dental Medicine, Yokohama 230-8501, Japan; yamamoto-y@tsurumi-u.ac.jp (Y.Y.); hosoya-n@tsurumi-u.ac.jp (N.H.); 5 Department of Translational Research, Tsurumi University School of Dental Medicine, Yokohama 230-8501, Japan; hanada-n@tsurumi-u.ac.jp

**Keywords:** dental hygienist, job satisfaction, career retention, daily tasks, working environments

## Abstract

In Japan, there is currently a shortage of dental hygienists. The number of dental hygienists as a workforce at dental clinical practice is not sufficient. Several factors affect career retention and job satisfaction of hygienists and these factors are considered to correlate with each other to construct networks. The aim of this study was to present a structural model of job satisfaction of Japanese dental hygienists and to determine the characteristics of unmotivated hygienists. The Japan Dental Hygienists’ Association has conducted a survey on their working environments every five years since 1981. Questionnaires were sent to all members of the association (16,113) and 8932 answers were returned. The data of 3807 active dental hygienists who worked at clinics were analyzed. Items associated with job satisfaction were derived from two latent variables, namely, the intrinsic psychosocial factors for the value of the work and extrinsic employment advantage. Based on the structural equation modeling, the association of value was higher than that of advantage. Most of the hygienists wished to continue working as dental hygienists. More than 60% felt their work required a high level of expertise. The value of the profession is deeply rooted in job satisfaction, motivation, and job retention of Japanese dental hygienists. Working environments where dental hygienists make great use of their specialized skills can lead to high career retention which prevent them from taking career breaks.

## 1. Introduction

The Japanese Dental Hygienists Law states that a dental hygienist’s duty is to improve oral health status [1]. To accomplish this, Japanese dental hygienists carry out oral hygiene care, oral hygiene instruction, drug application to the tooth and mucosal surfaces, mechanical removal of tooth surface deposits on the gingival margin, and assisting dentists in clinical dental treatment under the supervision of dentists. In addition, in the rapidly aging Japanese society, there is an increasing demand in older adults for oral health managements, including routine professional oral care and oral function improvement training by dental hygienists.

Based on a national survey in Japan, 132,629 dental hygienists worked in 2018 [2]. The number of dental hygienists has been slightly increasing year by year: 96,442 in 2008, 103,180 in 2010, 108,123 in 2012, 116,299 in 2014, 123,831 in 2016, and 132,629 in 2018. In spite of the annual increase in numbers of dental hygienists, there is still a shortage to meet the needs of the Japanese population.

The dental service in Japan is sustained mainly by private clinics. As of 2018, there were 68,506 dental clinics in Japan [3]. A total of 12,211 (90.5%) dental hygienists worked in clinics [4]. The number of active dental hygienists per one clinic was only 1.6. It is impossible to provide appropriate dental hygiene care to patients in addition to regular services. Most dental hygienists in Japan are women. There are major life events as turning points for women: marriage, childbirth, and child-rearing. Working beneficial systems are usually not utilized for dental hygienists because private dental clinics do not have good benefits and welfare systems compared to hospitals with many employees [5,6,7]. These situations can lead dental hygienists to take career breaks from work or ultimately leave a job [8,9,10]. In Japan, once an employed hygienist leaves, he/she is not entitled to return to the same job unless the employment contract stipulates otherwise, which is a crucial problem that must be handled in an appropriate manner.

Several studies on the working environments of dental hygienists have been reported [5,6]. Career retention was affected by some factors: salary [9,10,11], family matters [9,10,11,12], boredom and lack of benefits [9,13], and psychological matters [9,11,12]. Dental hygienists who have left their jobs demanded better income [9,12], social benefits [13,14], or part-time work or flexibility in working hours [9,10]. Insufficient skills are a barrier to returning to jobs [4]. Forty-one percent of dental hygienists leaving their jobs have participated in a postgraduate training course or training workshops [13,14]. Many dental hygienists who have taken a career break have continued their education [14]. Dental hygienists working other than clinics are satisfied with their employment [15]. Therefore, improved working conditions should make the career break period shortened and prevent resignation.

Job satisfaction is important preconditions in continuing employments, and its levels are affected by many factors [16]. These factors constitute a complex network [17]. Increasing job satisfaction is indispensable to maintaining the quality of medical care quality [18]. The employment status and working conditions of healthcare workers vary between countries. However, most studies of healthcare workers’ job satisfaction focus on the same career retention factors: education or training [14,19,20,21], income [7,22,23], working environment and psychosocial factors [23,24,25,26], personal life [22], job security [22], task range [21,27], employment policies [7], and workload [25]. These factors are all associated with the job satisfaction of dental hygienists. Therefore, the Japan Dental Hygienists’ Association has conducted surveys to determine the effects of these factors on job satisfaction based on the previous report described above with some modifications.

These factors that affect job satisfaction correlate with one another and construct networks. Most previous studies applied descriptive statistics to determine the factors that represent job satisfaction. To elucidate the intercorrelations of these factors and the structure of the networks, statistical modeling is indispensable. Several studies have reported that data was analyzed using many statistical models. Especially, these models were often used in the field of nursing [18,28,29,30,31,32,33]. Based on these models, effective workforce plans can be implemented. However, only one structural model is available for dental hygienists [34]. Besides improvements in job satisfaction, understanding the factors that lead to dental hygienists leaving their jobs is significant; however, there is no report from this perspective.

In this study, we analyzed the data concerning job satisfaction collected in the Employment Status of Japanese Dental Hygienists survey conducted by the Japan Dental Hygienists’ Association. This study aimed to find out characteristics of the factors related to job satisfaction of the hygienists and present a structural model of job satisfaction. Additionally, we aimed to determine the characteristics of unmotivated dental hygienists.

## 2. Materials and Methods

### 2.1. Survey Method

The Japan Dental Hygienists’ Association has been conducting surveys of the employment status of Japanese dental hygienists every five years since 1981. Questionnaires were distributed to all 16,113 members of the Japan Dental Hygienists’ Association on 30 September 2014 by post, including a stamped addressed envelope for recovery. The survey execution date was set as 31 October [5,9,35]. The questionnaires that were returned by 30 November were used for the analysis.

### 2.2. Questionnaire

The questionnaire used in this study consisted of 94 items concerning demographic factors, employment situation, working contents, willingness to work, etc. Many of these items were based on the order of the Ministry of Health, Labor, and Welfare to grasp the whole picture of the working conditions of dental hygienists [5,6,8,9,35]. The Japan Dental Hygienists’ Association included the items concerning the job satisfaction and willingness to work of dental hygienists.

The items analyzed in this study were two items concerning the will of Japanese dental hygienists: “Do you feel that dental hygienist work is valuable?” and “Do you wish to continue working as a dental hygienist?” Eight other items with regard to attractiveness of dental hygienist work were selected for analysis: “Do you think that dental hygienist work is to maintain people’s health?”, “Do you think that dental hygienist work can contribute to people and the community?”, “Do you think that being a dental hygienist is good to yourself?”, “Do you think that dental hygienist work requires a high level of expertise?”, “Do you think that dental hygienist work provides employment security?” , “Do you think that income is assured?”, “Do you think that only nationally licensed dental hygienists are employed as dental hygienists?” and “Do you think that women are better suited to working as dental hygienists?” The questionnaire analyzed in this study is presented in Table S1 (Supplementary Materials).

### 2.3. Statistical Analysis

Descriptive statistics and cross-tabulations with age groups were summarized for the questions described above. Chi-square tests were used to find out the statistical significance. Factor analysis with varimax rotation was carried out to create the construct for structural equation modeling. The structural relationship between the will of Japanese dental hygienists and the attractiveness of hygienist work was calculated by structural equation (SEM), which can summarize the observed variables by latent variables and analyze the relationships of latent variables. Analysis was performed by AMOS software (AMOS version 24.0, IBM, Tokyo, Japan).

Item response theory (IRT) was applied to calculate the item discriminations and the item difficulties pertaining to the attractiveness of hygienist work. Item response curves and item information curves were graphically illustrated. IRT analysis were carried out by R version 3.50 with the irtoys package using the following equation:

(1) $$\left(U_{i,j,}|\theta_{i},a_{j},b_{j}\right)=\frac{exp\left(1.76a_{j}\left(\theta_{i}-b_{j}\right)\right)}{1+exp\left(1.76a_{j}\left(\theta_{i}-b_{j}\right)\right)}$$

Decision analysis was carried out using classification and regression trees (CARTs). Two items concerning the will of Japanese dental hygienists were used for the objective variables, and eight items for the attractiveness of dental hygienist work were used for the explanatory variables. CART uses Gini Impurity for splitting the dataset into a decision tree. From the root node, the item that had the highest Gini Impurity was selected as the leaf node. The same procedure was then repeated. SPSS Statistics version 24.0 (IBM, Tokyo, Japan) was used for analysis [36,37,38,39].

### 2.4. Ethics

This study was approved by the Ethics Committee of Tsurumi University School of Dental Medicine (approval number: 1837) and conducted in accordance with the Declaration of Helsinki.

## 3. Results

### 3.1. Descriptive Statistics of the Survey

A total of 16,113 questionnaires were sent to all members of the Japan Dental Hygienists’ Association, and we received 8932 answers: the number of valid responses was 8932, resulting in a response rate of 53.4%. The number of hygienists leaving their jobs was 1350 (15.1%), while the number of hygienists working in dental clinics was 3807 (42.6%), 1096 (12.3%) in hospitals, 989 (11.1%) in government facilities, 472 (5.3%) in dental hygiene schools, 326 (3.7%) in nursing homes, and 892 (10.0%) in other places. The data of the hygienists working in dental clinics were used for the following analysis.

### 3.2. Descriptive Statistics of the Items That Affect the Will of Dental Hygienist Work

For the eight items that may contribute to the job satisfaction of hygienists, cross-tabulations against “Do you wish to continue working as a dental hygienist?” and “Do you feel that dental hygienist work is rewarding?” were calculated. The results are shown in Table S2 (Supplementary Materials). The item “Do you think that dental hygienist work provides employment security?” was not statistically significant for either “Do you wish to continue working as a dental hygienist?” or “Do you feel that the dental hygienist work is rewarding?” The seven other factors were statistically significant.

### 3.3. Characteristics of the Items That Affect the Will of Dental Hygienists’ Work

To investigate the structure of the eight items that contribute to job satisfaction, factor analysis using the likelihood method with varimax rotation was carried out. The results are shown in Table S3 (Supplementary Materials). The items were clearly divided into two factors, and these latent variables were named as “value” and “advantage”.

**Figure 1:**
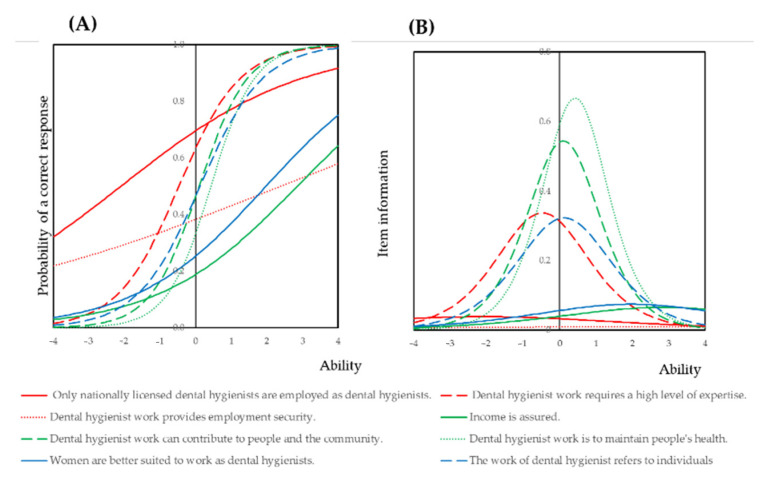
Item response and item information curves of the eight items contributing to the job satisfaction of dental hygienists. (**A**) Item response curve. (**B**) Item information curve.

IRT analysis was carried out to elucidate the response for these eight items. The results of the two-parameter logistic model are shown in Table S4 (Supplementary Materials). The item discriminations of value were higher than those of advantage. The item response and item information curves are illustrated in Figure 1. The item response curves concerning value show clear sigmoid curves. In contrast, the curves concerned with advantage are gently sloping.

The eight items that affect the job satisfaction of dental hygienist work were analyzed by a two-parameter logistic model of item response theory (IRT). The item response curves derived from the latent variable “value” illustrate sigmoid curves, while those derived from the latent variable “advantage” display gentle slopes. The horizontal axis, depicting the ability by IRT, indicates the level of job satisfaction by the weighted total number of “yes” answered to the items. The vertical axis indicates the percentage of dental hygienists who answered “yes” to the factors associated with job satisfaction. From the item information curve, items derived from value shown in Figure 1 had higher item information when compared to the items derived from advantage.

### 3.4. Structure of Motivation and Its Associated Items

**Figure 2:**
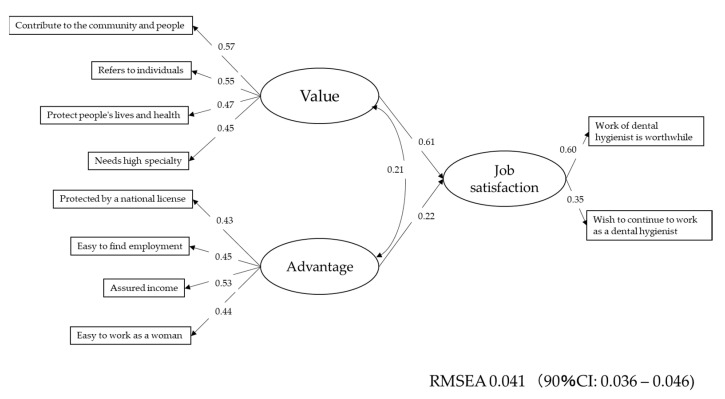
The path diagram of job satisfaction of dental hygienists. A path from “Value” to “Job satisfaction” was higher than that from “Advantage” to “Job satisfaction.” REMSEA stands for root mean square error of approximation.

By using this result, structural equation modeling (SEM) was carried out. The results are shown in Figure 2. A path from “Value” to “Job satisfaction” was higher than that from “Advantage.”

### 3.5. Determining the Perfunctory of Dental Hygienists

The number of dental hygienists who answered “strongly disagree” for the item “Do you feel that dental hygienist work is rewarding?” was 42 (1.1%) and “no” for the item “Do you wish to continue working as a dental hygienist?” was 218 (5.8%). Even though these hygienists only made up a small minority, figuring out the number of hygienists who actually had negative answer to these items is important. A decision analysis to find out the rules for these dental hygienists was carried out.

**Figure 3:**
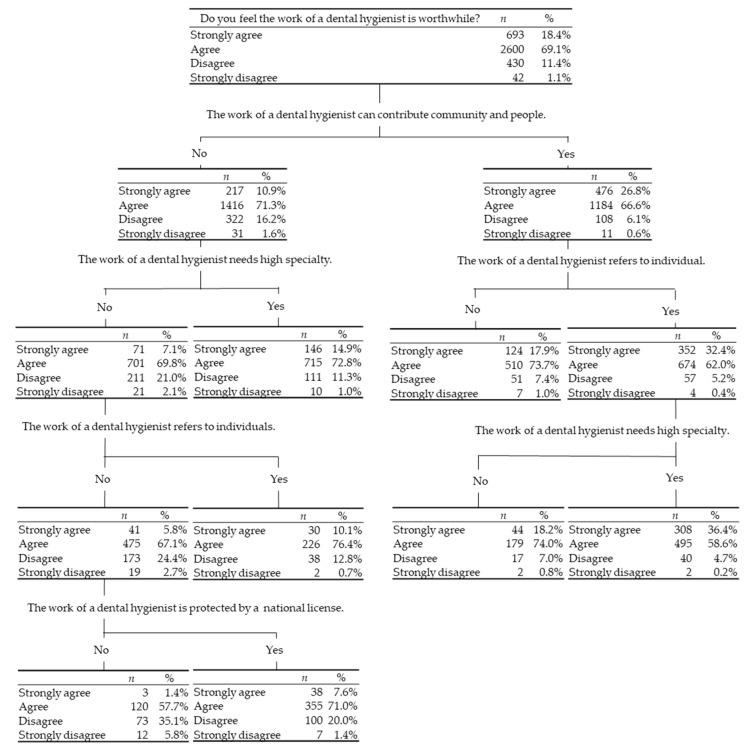
Decision analysis to determine the dental hygienists who answered “strongly disagree” to “Do you feel that dental hygienist work is rewarding?”

Based on the decision analysis, the response pattern of the minor but important fraction of clusters could be found. The subjects who answered “strongly disagree” to “Do you feel that dental hygienist work is rewarding?” was only 42 (1.1%). Determining the response pattern of these subjects may help improve the quality of policy-making, education, and their working environment. Among the 42 hygienists, 31 (73.8%) answered “no” to “Do you think that dental hygienist work can contribute to people and the community?” and 21 (50%) answered “no” to “Do you think that dental hygienist work requires a high level of expertise?” and 19 (45.2%) answered “no” to “Do you think that being a dental hygienist is good to yourself?” Finally, 12 (28.6%) answered “no” to “Do you think that only nationally licensed dental hygienists are employed to work as dental hygienists?” Therefore, of all the hygienists who feel that their work is not rewarding, 28.6% answered “no” to all of the four items discussed above (Figure 3).

**Figure 4:**
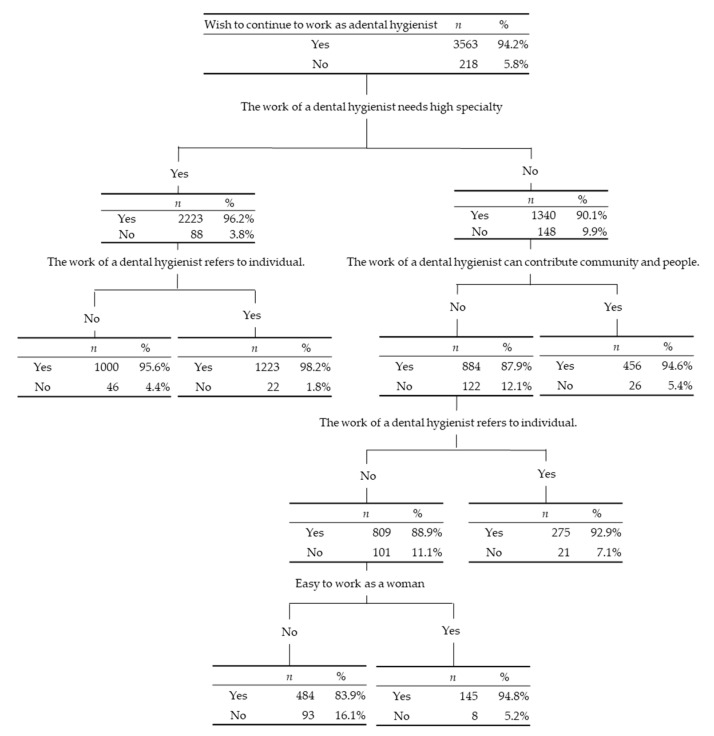
Decision analysis to determine the dental hygienists who answered “no” to “Do you wish to continue working as a dental hygienist?”

Similarly, 218 dental hygienists did not wish to continue their work. Among them, 148 (67.9%) answered “no” to “Do you think that dental hygienist work requires a high level of expertise?” followed by 112 (51.4%) who answered “no” to “Do you think dental hygienist work can contribute to people and the community?”, 101 (46.3%) who answered “no” to “Do you think that being a dental hygienist is good to yourself?”, and 93 (42.7%) who answered “no” to “Do you think that women are better suited to working as dental hygienists?” Therefore, 93 (42.3%) of hygienists who did not wish to continue working as hygienists answered “no” to all of the four items described above (Figure 4).

## 4. Discussion

To the best of our knowledge, this is the first study to present a structural model of the job satisfaction of Japanese dental hygienists, and the characteristics of unmotivated dental hygienists were elucidated.

The items concerning job satisfaction were selected from a previous report, as described in the introduction. These items correlated with one another. Therefore, by factor analysis and SEM, the structural model presented in Figure 1 was constructed. The latent variable of advantage consisted of four items. The highest coefficient was the path from “Income is assured.” Several studies have suggested that income is the most important factor for job satisfaction and job retention for healthcare workers [40,41,42,43,44,45,46,47,48,49,50] and that this is applicable for dental hygienists as well [7,21,22]. In order to deter dental hygienists from leaving their jobs, it seemed to be necessary to take improvement measures not only for enhancing the attractiveness to work as dental hygienists but also for the stability of the reward. Considering that dental care in Japan is covered by public medical insurance, it may be necessary to take measures such as raising the remuneration of dental hygienists in order to improve salary aspects.

The latent variable of value consisted of five items. These items were intrinsic psychosocial factors. The highest co-efficient was “Do you think that dental hygienist work can contribute to people and the community?” followed by “Do you think that being a dental hygienist is good to yourself?” A previous report concerning maintaining the job satisfaction of nurses showed that intrinsic factors are more important than extrinsic factors [50]. The coefficient of the path from “Value” to “Job satisfaction” was higher than that of the path from “Advantage” to “Job satisfaction.” These internal factors may imply the value of the profession as a dental hygienist. The model presented in Figure 1 is consistent with this previous report. The value of the profession is embedded in the job satisfaction, motivation, and job retention of Japanese dental hygienists. The model presented in Figure 1 is the first model to analyze the factors that affect the job satisfaction of dental hygienists. However, the loadings by single factors were not high and thus psychometric properties may not enough. In addition, there is no model to compare the validity of the presented model in this study to be. Therefore, the items generally considered to be concerned with job satisfaction may not have enough psychometric properties, indicating that further study is necessary to construct more sophisticated models for analyzing the job satisfaction of dental hygienists.

Based on the item response curves shown in Figure 2, the items derived from the latent variable value displayed a parallel sigmoid curve pattern, and the item information curves of these items were higher than the items derived from latent variable advance. This indicates that the number of dental hygienists who answered “yes” increased as job satisfaction increased. In contrast, the items derived from latent variable advantage showed a gently sloping trend, and these items had low item information. These results indicate that the dental hygienists answered “yes” case by case, as the number of “yes” responses for these items did not increase proportionally.

For the item “Do you feel that dental hygienist work is rewarding?”, 42 dental hygienists answered “strongly disagree.” Based on the decision tree shown in Figure 3, 21 (50%) dental hygienists answered “no” to all four items derived from the latent variable value. In contrast, 693 dental hygienists answered “strongly agree” to “Do you feel that dental hygienist work is rewarding?” Among them, 308 (44.4%) answered “yes” to the three items derived from the latent variable value. Meanwhile, 218 (5.8%) hygienists did not wish to continue working as a dental hygienist. These subjects made up only a small fraction of the studied population, and they answered “no” to the three items derived from the latent variable value. Therefore, these factors should be improved to prevent hygienists from taking a career break. However, changing intrinsic psychosocial factors is not an easy task. Nagatani et al. pointed out that professionalism is multifaceted and shaped by academic backgrounds, clinical experiences, and social interactions in a qualitative study of Japanese dental hygienists [51]. The same things may apply to the value and jog satisfaction as dental hygienists, i.e., continued career support from dental hygienist students to post-qualification may increase their value as dental hygienists and lead to continuing employment. Education of the value of dental hygienists as healthcare workers may be important. In contrast, most of the dental hygienists wished to continue to work. Among them, 2223 (62.4%) felt their work requires a high level of expertise. A previous report showed that Japanese dental hygienists engage in the management and ordering of drugs and dental equipment (77.8%), as well as the sterilization and disinfection of dental equipment (91.6%) as daily tasks [35,52]. These menial tasks do not always make the most of their specialized skills which could possibly discourage their professional mindset; therefore, to stay motivated and prevent them from taking a career break, they should be engaged in the daily tasks that require their specialized skills.

## 5. Conclusions

Politeness is built into the job satisfaction, motivation, and job maintenance of Japanese dental hygienists. Dental hygienists should be engaged in daily, professional tasks that require their specialized skills which can prevent them from taking career breaks and leaving careers. Several limitations of the present study need to be addressed. Because the study participants were only dental hygienists who were members of Japan Dental Hygienists’ Association, the findings obtained from these self-reported data can only be generalized within this population. Since the range and characteristics of dental hygienists’ daily tasks are likely to vary from country to country, studies such as international comparisons will also be needed in the future. To understand the job satisfaction, items concerning organization culture, working temperature, leadership of organizations and relationship for each employee should be investigated. Low response rate of the questionnaire may be considered another limitation of this study.

## Data Availability

The data of the present study were used under license for the current study and, therefore, are not publicly available.

## Supplementary Materials

The following are available online at https://www.mdpi.com/1660-4601/18/6/3200/s1: Table S1: The items in the questionnaire analyzed in this study; Table S2. Cross-tabulations of the job satisfaction of Japanese dental hygienists and the associated factors; Table S3. Results of the factor analysis; Table S4. Results of the item response theory analysis by the two-parameter logistic model.
