# Supplementary material for: Impact of Working Environment on Job Satisfaction: Findings from a Survey of Japanese Dental Hygienists
Source: Int J Environ Res Public Health. 2021 Mar 19;18(6):3200. doi: 10.3390/ijerph18063200 (PMC8003819; doi:10.3390/ijerph18063200)
Supplement: Supplementary file 1 [file ijerph-18-03200-s001.pdf]

International Journal of
*Environmental Research and Public Health*

MDPI

**Table S1.** The items in the questionnaire analyzed in this study.

| | | |
|---|---|---|
| | **Please answer following items concerning volition of dental hygienists.** | |
| 1 | Do you feel that dental hygienist work is valuable? | Strongly agree<br>Agree<br>Disagree<br>Strongly disagree |
| 2 | Do you wish to continue working as a dental hygienist? | (Yes, No) |
| | **Please answer following items concerning attractiveness of dental hygienist work**. | |
| 1 | Do you think that dental hygienist work is to maintain people's health? | (Yes, No) |
| 2 | Do you think that dental hygienist work can contribute to people and the community? | (Yes, No) |
| 3 | Do you think that being a dental hygienist is good to yourself? | (Yes, No) |
| 4 | Do you think that dental hygienist work requires a high level of expertise? | (Yes, No) |
| 5 | Do you think that dental hygienist work provides employment security? | (Yes, No) |
| 6 | Do you think that income is assured? | (Yes, No) |
| 7 | Do you think that only nationally licensed dental hygienists are employed as dental hygienists? | (Yes, No) |
| 8 | Do you think that women are better suited to working as dental hygienists? | (Yes, No) |

**Table 2.** Cross tabulations of motivation of Japanese dental hygienist and its associated factors.

**(A) Wish to continue to work as dental hygienist**

| Attractiveness of dental hygienist work | | Wish to Continue to Work as Dental Hygienist: Yes | No | Total | p-Value |
|---|---|---|---|---|---|
| The work of dental hygienist is to protect people's lives and health. | No | 2165 | 171 | 2336 | <0.001 |
| | Yes | 1398 | 45 | 1443 | |
| The work of dental hygienist can contribute community and people. | No | 1830 | 165 | 1995 | <0.001 |
| | Yes | 1733 | 51 | 1784 | |
| The work of dental hygienist refers to individual. | No | 1835 | 161 | 1996 | <0.001 |
| | Yes | 1728 | 55 | 1783 | |
| The work of dental hygienist needs high specialty. | No | 1340 | 148 | 1488 | <0.001 |
| | Yes | 2223 | 68 | 2291 | |
| Not bothered by finding employment. | No | 2176 | 148 | 2324 | 0.029 |
| | Yes | 1387 | 68 | 1455 | |
| Income is assured. | No | 2829 | 193 | 3022 | <0.001 |
| | Yes | 734 | 23 | 757 | |
| The work of dental hygienist is protected by national license. | No | 1054 | 117 | 1171 | <0.001 |
| | Yes | 2509 | 99 | 2608 | |
| Easy to work as a woman. | No | 2585 | 188 | 2773 | <0.001 |
| | Yes | 978 | 28 | 1006 | |

**(B) Work of dental hygienist is valuable**

| Attractiveness of dental hygienist work | | Do You Feel the Work of Dental Hygienist Is Valuable?: Strongly agree | Agree | Disagree | Strongly disagree | Total | p-Value |
|---|---|---|---|---|---|---|---|
| The work of dental hygienist is to protect people's lives and health. | No | 306 | 1670 | 320 | 32 | 2328 | <0.00 1 |
| | Yes | 387 | 930 | 110 | 10 | 1437 | |
| The work of dental hygienist can contribute community and people. | No | 217 | 1416 | 322 | 31 | 1986 | <0.00 1 |
| | Yes | 476 | 1184 | 108 | 11 | 1779 | |
| The work of dental hygienist refers to individual. | No | 234 | 1431 | 295 | 32 | 1992 | <0.00 1 |
| | Yes | 459 | 1169 | 135 | 10 | 1773 | |
| The work of dental hygienist needs high specialty. | No | 146 | 1059 | 256 | 25 | 1486 | <0.00 1 |
| | Yes | 547 | 1541 | 174 | 17 | 2279 | |
| Not bothered by finding employment. | No | 433 | 1579 | 272 | 29 | 2313 | 0.476 |
| | Yes | 260 | 1021 | 158 | 13 | 1452 | |
| Income is assured. | No | 492 | 2102 | 378 | 37 | 3009 | <0.00 1 |
| | Yes | 201 | 498 | 52 | 5 | 756 | |
| The work of dental hygienist is protected by national license. | No | 145 | 805 | 194 | 19 | 1163 | <0.00 1 |
| | Yes | 548 | 1795 | 236 | 23 | 2602 | |
| Easy to work as a woman. | No | 459 | 1914 | 353 | 36 | 2762 | <0.00 1 |
| | Yes | 234 | 686 | 77 | 6 | 1003 | |

**Table S3:** Results of factor analysis.

| **Attractiveness of dental hygienist work** | **Factor** | |
|---|---|---|
| | **1** | **2** |
| The work of dental hygienist is to protect people's lives and health. | 0.58 | 0.05 |
| The work of dental hygienist can contribute community and people. | 0.56 | 0.04 |
| The work of dental hygienist refers to individual. | 0.48 | 0.02 |
| The work of dental hygienist needs high specialty. | 0.41 | 0.12 |
| Not bothered by finding employment. | −0.05 | 0.51 |
| Income is assured. | 0.07 | 0.50 |
| The work of dental hygienist is protected by national license. | 0.06 | 0.42 |
| Easy to work as a woman. | 0.13 | 0.41 |
| Total | 1.08 | 0.87 |
| Variance% | 13.45 | 10.90 |
| Cumulative variance% | 13.45 | 24.35 |

Factor analysis was carried out for the eight items that concerns with the volition of dental hygienist. Factor analysis was carried out with Varimax rotation.

**Table S4.** Results of Item response theory analysis by two parameter logistic model.

| attractiveness of dental hygienist work | Item discrimination ($a_j$) | | Item difficulty ($b_j$) | |
|---|---|---|---|---|
| | Estimate | SE | Estimate | SE |
| The work of dental hygienist is protected by national license. | 0.39 | 0.05 | −2.11 | 0.28 |
| The work of dental hygienist needs high specialty. | 1.16 | 0.08 | −0.47 | 0.04 |
| Not bothered by finding employment. | 0.20 | 0.05 | 2.39 | 0.59 |
| Income is assured. | 0.51 | 0.06 | 2.85 | 0.32 |
| The work of dental hygienist can contribute community and people. | 1.47 | 0.10 | 0.10 | 0.03 |
| The work of dental hygienist is to protect people's lives and health. | 1.63 | 0.11 | 0.43 | 0.03 |
| Easy to work as a woman. | 0.55 | 0.06 | 1.98 | 0.20 |
| The work of dental hygienist refers to individual. | 1.14 | 0.07 | 0.12 | 0.04 |
